# Phosphorus Release from Nano-Hydroxyapatite Derived from Biowastes in the Presence of Phosphate-Solubilizing Bacteria: A Soil Column Experiment

**DOI:** 10.1021/acs.jafc.4c09325

**Published:** 2025-02-07

**Authors:** Laura Pilotto, Francesca Scalera, Clara Piccirillo, Luca Marchiol, Monica Yorlady Alzate Zuluaga, Youry Pii, Stefano Cesco, Marcello Civilini, Guido Fellet

**Affiliations:** † Department of Life Sciences, University of Trieste, via Licio Giorgieri 10, Trieste 34127, Italy; ‡ Department of Agrifood, Environmental and Animal Sciences, University of Udine, via delle Scienze 206, Udine 33100, Italy; § Institute of Nanotechnology CNR-NANOTEC, Campus Ecotekne, Via Monteroni 165, Lecce 73100, Italy; ∥ Faculty of Science and Technology, Free University of Bozen/Bolzano, Universitätsplatz 5 - Piazza Università, 5, Bolzano 39100, Italy

**Keywords:** food wastes, nHAP, nanoenabled agriculture, *P. alloputida*, P losses, soil column experiment

## Abstract

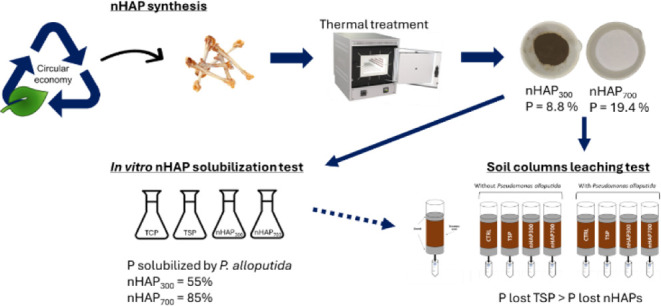

Phosphorus applications in agriculture can lead to significant environmental impacts, necessitating a revolution in current agricultural practices. This study explores the potential of hydroxyapatite nanoparticles (nHAPs) synthesized from poultry bones as P fertilizers. nHAPs were produced at 300 °C (nHAP_300_) and 700 °C (nHAP_700_), and their effectiveness was evaluated. An *in vitro* solubilization test with *Pseudomonas alloputida* evaluated the bacterium’s ability to solubilize the nanoparticles, assessing dissolved P and organic acids produced. Additionally, a soil leaching test measured P losses and bioavailable P in soil compared to a conventional fertilizer, the triple superphosphate (TSP). nHAP_300_ displayed heterogeneous sizes, while nHAP_700_ were approximately 100 nm in size, with a P content of 8.8% and 19.4%, respectively. *Pseudomonas alloputida* successfully solubilized both types of nanoparticles, with nHAP_700_ demonstrating a higher solubility than nHAP_300_. The TSP treatment resulted in higher P losses (6.35 mg) compared with nHAP treatments (nHAP_300_ 0.32 mg; nHAP_700_ 0.28 mg), indicating the potential of nHAP for recycling P from waste. Our findings indicate that nHAP_700_ are more efficient in P release than nHAP_300_ but less prone to leaching compared to conventional fertilizers. Utilizing these nanoparticles enables phosphorus recovery from waste and holds significant potential for sustainable agricultural applications.

## 1 Introduction

Managing soil fertility is a crucial goal for achieving sustainable agriculture.^1,2^ At present, synthetic fertilizers are widely used but exhibit high inefficiency.^3^ This leads to substantial nutrient loss, causing negative environmental effects, as well as energy wastage, and depletion of financial resources.^4,5^ Concerning crop phosphorus (P) nutrition, commercial P fertilizers exhibit a nutrient use efficiency (NUE) of about 15–30%,^6^ thus indicating that only a fraction of the applied nutrients is absorbed by plants in the year of application. The remaining phosphorus either becomes bound to the soil or is lost, necessitating farmers to increase fertilizer rates. However, this escalation has resulted in detrimental effects on freshwater and groundwater quality.^7^ In fact, P is primarily retained in soils through adsorption, but this capacity is limited; once exceeded, excess P can dissolve and move with water through runoff or leaching into groundwater, contributing to contamination of water bodies and long-term environmental impacts from overapplication of fertilizers and manure.^8^ P, together with nitrogen, is one of the key factors in the eutrophication of water bodies,^9^ which is estimated to impact 1.7 billion people, and the costs of addressing the issue are considerable, with annual expenditures estimated at $1 billion in Europe and $2.4 billion in the United States.^10^ Furthermore, P can reach the groundwater and potentially exceed legal safety thresholds, posing risks to water quality and public health.^8^

Recognizing the imperative for sustainable crop fertilization, significant efforts are required to comprehend the principles governing it. Enhancing nutrient uptake and use efficiency, employing green fertilizers and pesticides judiciously, and establishing sustainable agricultural systems are all pivotal components of modern agriculture.^11^ Nanoenabled agriculture has ignited research interest in recent years.^12^ Ongoing studies explore the potential of smart nanostructures for targeted release and distribution of nutrients, agrochemicals, and biomolecules. Anticipated advancements include intelligent control of nutrient release based on plant developmental stages, alongside the utilization of nanomaterials in plant nutrition.^13^

Kopittke et al. (2019) stated that various nanomaterials can be used as fertilizers in agriculture. These include both nanomaterials composed of the nutrient to be delivered and those that are carriers of the desired nutrient(s). Recently, significant attention has been given to hydroxyapatite nanocrystals.^14^ Hydroxyapatite (HAP, Ca_10_(PO_4_)_6_(OH_2_)) with a Ca/P molar ratio of 1.67 can be extracted from biological sources and wastes, such as bovine and horse bones, fish bones, and scales.^15^ The calcium phosphate derived from dried bones is classified as biogenic crystalline apatite and is slightly more soluble than geological apatite found in rock phosphates.^16^ Moreover, compared to the stoichiometric hydroxyapatite, the biogenic one contains impurities, such as Na^+^, Zn^2+^, Mg^2+^, K^+^, Si^2+^, and CO_3_^2−^.^17^ These are elements and/or ions generally present in the bones; consequently, they are also found in the hydroxyapatite derived from them.^18^ Notably, these impurities can serve as nutrients for plants.^19^

The properties of hydroxyapatite at the nanoscale (nanohydroxyapatite, nHAP) present promising applications in agriculture, including its use as a phosphorus source for crops, but it has also been applied as a carrier of other elements or molecules helpful for plant nutrition and protection.^20^ A literature review was recently conducted to assess the progress of scientific research on the use of crystalline nHAPs as crop fertilizers. The findings underscore the promising potential of nHAP, either alone or in combination with other molecules, as a more efficient alternative to conventional N and P fertilizers.^21^ The use of this nanomaterial can enable the slow release of P,^22^ thus prolonging the persistence of the nutrient in the agro-ecosystem. This feature would provide crops with optimal nutrient levels over an extended period, ultimately resulting^22,23^ in increased biomass and improved P content in cultivated plants.^21^ At the same time, another review identified several critical challenges in nHAP application, including inconsistencies in experimental methods and variations in model species, soil types, and nanoparticle synthesis techniques, which lead to differences in size and properties and thus to different results.^20^ In any case, despite its potential, the low solubility of nHAP remains a significant limitation for its effectiveness in improving plant phosphate nutrition.^24^

Current research is focusing on addressing this low solubility through various approaches, including slow-release P fertilizer and the use of phosphate-solubilizing bacteria (PSB) or arbuscular mycorrhizal fungi (AMF) that enhance P availability and serve as carriers for both macro- and micronutrients. PSB, commonly found in bulk soil and the rhizosphere of most plants, are of particular interest due to their significant role in improving P nutrition, development, and yields in crops.^25^ Documented genera, such as *Pseudomonas*,^26,27^*Bacillus*,^28^*Enterobacter*,^25,29^*Burkholderia*,^30^*Pantoea*,^31^ and *Acinetobacter*,^32^ illustrate the breadth of PSB species effective in enhancing P solubilization. One key mechanism employed by *Pseudomonas* and other PSB for mineral P solubilization is the release of organic acids (OAs) and protons.^33,34^ Indeed, the release of protons lowers the pH of the rhizosphere, creating an acidic environment that promotes the desorption of P from mineral surfaces.^33^ Concurrently, the OAs produced by PSB, thanks to their carboxyl and hydroxyl functional groups, play crucial roles in ligand exchange reactions. These functional groups have a high affinity for metal cations such as Ca^2+^, Fe^3+^, and Al^3+^, which are commonly associated with P in soil minerals.^29,35^ The carboxyl groups can displace phosphate ions from metal–P complexes via complexation, effectively breaking down the mineral structures that limit P availability.^36^ Additionally, OAs contribute to P mobilization by chelating these cations, thereby weakening their interaction with phosphate ions and enhancing the release of P into the soil solution, where it becomes more readily available for plant uptake.^37^

In line with circular economy principles, this work aims to test nHAPs from animal waste, specifically chicken bones, for the controlled release of P for plants using phosphate-solubilizing bacteria. This approach allows for the optimization of sustainable natural resource management and the valorization of slaughterhouse waste, particularly bones, for calcium phosphate production. The goal is to significantly reduce the use of nonrenewable P sources while concurrently preserving soil fertility, especially concerning phosphorus.

nHAPs were prepared from chicken bones with thermal treatment at two different temperatures (300 and 700 °C), and the obtained powders were fully characterized. Their solubilization was tested in solution with a PSB, namely *Pseudomonas alloputida*. A soil-leaching experiment was also performed to test the behavior of these materials compared to the traditional fertilizer triple superphosphate (TSP) in soil columns with and without the PSB strain.

## 2 Materials and Methods

### 2.1 nHAP Synthesis and Characterization

Chicken bones were supplied by a local butcher. First, the bones were boiled in distilled water for 1 h and washed to eliminate as much organic and protein material as possible. The raw waste materials were then heated for 1 h in a furnace with atmospheric air at two different temperatures, 300 °C (nHAP_300_) and 700 °C (nHAP_700_). In both cases, the thermal ramp was 5 °C min^–1^. The resulting products were initially ground manually in a mortar; successively, they were further ground with an automatic mortar for 3 h to obtain powdery materials with smaller and more uniform particle sizes. The morphology of biowaste materials was analyzed by Scanning Electron Microscopy (SEM) using a Carl Zeiss Merlin instrument, equipped with a Gemini II column and an integrated high-efficiency In-lens for secondary electrons (Carl Zeiss, Oberkochen, Germany). Samples were sputtered with gold before the analysis. Transmission Electron Microscopy (TEM) analyses were performed using a JEOL-Jem 1011 microscope, working at an accelerating voltage of 100 kV. The samples were prepared by dropping a dilute solution of each sample on 400-mesh carbon-coated Cu grids and then dried at room temperature. The powders were analyzed with Fourier Transform Infrared Spectroscopy (FTIR) using an FT/IR-6000 Jasco spectrometer (Jasco Europe, Lecco, Italy); the spectra were acquired in the interval between 500 and 4000 cm^–1^, in transmittance mode. To do this, about 2 mg of powder was mixed with 200 mg of KBr to make a pellet, which was then used for the analysis. The phase composition, crystallinity, and crystallite size of the nanopowders were analyzed via X-ray diffraction (XRD) using an X’Pert PRO MRD diffractometer (Malvern Panalytical, Malvern, UK) with a fast RTMS detector and CuKα radiation (40 kV, 40 mA). Data were recorded in the 20–60° 2θ range with a 0.02° virtual step-scan and 200 s virtual time-per-step. Phase identification utilized JCPDS standards, specifically card 09-0432 for HAP and N09-169 for β-TCP. The fraction of crystalline phase χ_c_ present in the HAP powders was calculated using the following relation:^38^
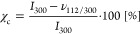
1where *I*_300_ is the intensity of the (300) reflection and *v* is the intensity of the hollow between (112) and (300) reflections.

The crystallite size (in nm) was estimated according to Scherrer’s equation:
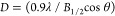
2where λ is the wavelength of the CuKα radiation (0.15406 nm), *B* is the full width at half-maximum (fwhm) peak intensity (radian), and θ is the Bragg diffraction angle (in degrees).

Elemental content was determined by an ICP-OES (Agilent 5800, Santa Clara, CA, USA), following digestion in a microwave oven (Milestone Ethos Easy, Bergamo, Italy) adopting the US EPA 3052 protocol with some modifications.^39^ Briefly, 100 mg of nanoparticles were digested in 9 mL of HNO_3_ and 1 mL of H_2_O_2_. Times and temperatures followed the protocol. Carbon and nitrogen contents were obtained with a CHN Analyzer (Elemental Vario El Cube, Langenselbold, Germany).

The surface area of the samples was measured by using a Micrometrics TriStar II Plus instrument; samples were degassed at 100 °C prior to the analysis. From the BET surface area, the size of the powder was estimated according to the following equation:^38^
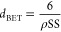
3where ρ is the density of the powder, and SS stands for its surface area. A density value of 3.156 g·cm^–3^, which is the theoretical density of stoichiometric hydroxyapatite, was used.

Moreover, their surface charge was measured with Malvern Nano-ZS90 equipment. The measurements were performed with a 1 g·L^–1^ suspension in distilled water, as well as in Luria–Bertani and NBRIP growth media, as these were employed for the tests with the bacterial strain (see the following section).

### 2.2 Bacterial Inoculant

*Pseudomonas alloputida* strain DSM 6125 was selected for its potential ability in solubilizing tricalcium phosphate (TCP, Ca_3_(PO_4_)_2_). The bacterial strain was grown in Luria–Bertani (LB) medium^40^ under orbital shaking (MaxQ 8000, Thermo Scientific, Germany) at 180 rpm, 28 °C for 48 h. Afterward, cells were harvested by centrifugation (SL16R, Thermo Scientific, Germany) at 5000× g for 10 min, washed, and resuspended in a sterile saline solution (0.85% w/v NaCl). The bacterial population density was quantified by performing direct counts using a Neubauer chamber^41^ and subsequently adjusted to a standard concentration of 10^8^ cell·mL^–1^ that was used as the inoculant for the following experiments.^42^

### 2.3 Determination of the Phosphate and nHAP Solubilization Ability of *P. alloputida*

Four phosphate sources, (i) TCP (in powder, Thermo Fisher Scientific, Waltham, MA, USA), (ii) TSP (triple superphosphate, TSP, P_2_O_5_ = 46%, commercial product in granules, provided by Panfertil), (iii) nHAP_300_, and (iv) nHAP_700_, were used to evaluate the ability of *P. alloputida* in solubilizing inorganic phosphate. The experiment was performed as previously described.^29^ Briefly, 1 L Erlenmeyer flasks containing 300 mL of NBRIP medium^43^ were supplemented with the different phosphate sources (0.1% P (w/v), pH 7.0) and autoclaved at 121 °C, 1 atm, for 20 min. The bacterial strain was inoculated at a final concentration of 10^6^ cell·mL^–1^ in triplicate and incubated at 28 °C with shaking at 180 rpm for 7 days. A triplicate of uninoculated flasks was used as a negative control for each P source. Aliquots from each culture were collected before the inoculation (0 days) and every 24 h for the subsequent 7 days. The samples were centrifuged to pellet the cells (5000× g for 10 min, SL16R, Thermo Scientific, Germany) and filtered through a 0.22 μm Millipore filter to remove any residual cell particles. The supernatant was collected and used to quantify the solubilized P and pH values at each time point. Organic acid production and identification were also determined at each incubation point according to Zuluaga et al. (2023)^29^ by using HPLC on a cation exclusion column (Aminex HPX-87H 300 mm × 7.8 mm, Bio-Rad Laboratories Inc.).

### 2.4 Soil Column Leaching Test

For this part of the experiment, 32 PVC tubes (diameter = 4.30 cm, length = 30 cm) filled with 2 mm sieved soil mixed with 10% (w/w) sand were used. The soil was collected at the University of Udine Experimental Farm “A. Servadei” (Udine, Italy). The soil had the following texture: Clay = 22%; Silt = 24%; Sand = 54% and previously supported the growth of the region’s typical crops (rotation of wheat, maize, and forage). Table 1 reports soil characteristics and the analysis methods employed.

**Table 1:** Main Soil Characteristics and Respective Analysis Methodologies Applied

Soil properties		Methodology
Texture	Clay: 22%; Silt: 24%; Sand: 54%	Miller and Horneck, 2013^46^
pH	7.6 ± 0.1	FAO, 2021^47^
EC	434 ± 0.2 μS·cm^–1^	Sonnevelt and Van den Ende, 1971^48^
CEC	30.07 ± 0.76 cmol·kg^–1^	ISO 11260:2018^49^
Total P	1033.79 ± 27.23 mg·kg^–1^	US EPA 3051A – ICP-OES^50^
Total Fe	24342.5 ± 430.45 mg·kg^–1^	US EPA 3051A – ICP-OES^50^
Total Al	27506.87 ± 768.48 mg·kg^–1^	US EPA 3051A – ICP-OES^50^
Total Ca	30855.95 ± 430.45 mg·kg^–1^	US EPA 3051A – ICP-OES^50^
Olsen P	44.7 ± 0.40 mg·kg^–1^	FAO, 2021^45^

Four treatments were applied: control untreated (CTRL), conventional P fertilizer (triple superphosphate, TSP, P_2_O_5_ = 46%, commercial product in granules, provided by Panfertil), nHAP_300_, and nHAP_700_. TCP was not considered as a treatment in this experimental section, as the aim was to compare the two nanopowders with conventional fertilizer. In columns treated with phosphorus, an amount equivalent to 200 mg of P from either nanoparticles or conventional fertilizer was evenly mixed with the soil based on P content determined through ICP-OES (Agilent 5800, Santa Clara, CA, USA) analysis of the nanomaterials. Each treatment was also repeated in soil inoculated with the microorganism in order to evaluate the ability of *Pseudomonas alloputida* (PSB+) to solubilize nHAP in soil, for a total of eight treatments: (i) CTRL PSB–, (ii) TSP PSB–, (iii) nHAP_300_ PSB–, (iv) nHAP_700_ PSB–, (v) CTRL PSB+, (vi) TSP PSB+, (vii) nHAP_300_ PSB+, and (viii) nHAP_700_ PSB+. The soil used was previously sterilized in an autoclave at 121 °C for 1 h. The columns were set up with three layers, divided as follows: (i) 80 g of sterile sand as the bottom layer; (ii) the equivalent of 300 g of air-dried sterile uninoculated/inoculated soil, added in 10% of sand and amended with nanoparticles or P conventional fertilizer; (iii) 40 g of sterile sand for the upper layer. Four replicates for treatment were set up for a total of 32 soil columns. Each column was equipped at the bottom with a sheet of geotextile and a funnel to allow the leachates’ collection. 50 mL of ultrapure water was added to each column at the end of the setup to restore field capacity. Every 7 days, all columns were leached with 50 mL of sterile ultrapure water and then sealed with punctured parafilm to minimize microbial contamination. The first leaching was performed after a week. The column leachates were collected after 24 h, mineralized (Milestone Ethos Easy, Bergamo, Italy) following the USEPA 3015A^44^ protocol, and characterized for their P content with an ICP-OES. In total, seven waterings were carried out. At the end of the experiment, the bioavailable P of the soil was determined for each replicate, applying the Olsen-P method.^45^

### 2.5 Data Analysis

Statistical analysis was carried out using R 4.2.1 and RStudio 2023.09.1+494 software. Two-way ANOVA and the Tukey’s posthoc test at *p* ≤ 0.05 were carried out. Outliers were removed through the application of Dixon’s Test. Furthermore, several *t* tests were run for the pairwise comparison among the presence of the microorganism in the same treatment. The relationship between organic acid profiles and solubilization efficiency was evaluated with Pearson correlation analysis, using the *corrplot* package.

## 3 Results

### 3.1 Characterization of the nHAP Powders

**Figure 1:**
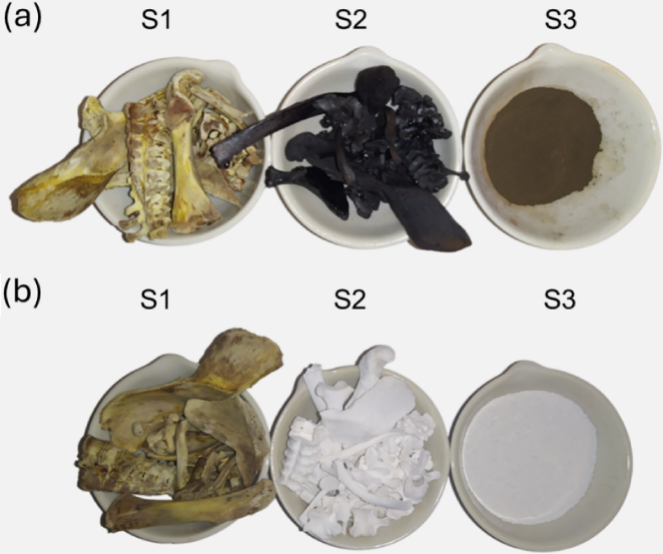
Steps of the biogenic nanohydroxyapatite synthesis. S1) Raw material; S2) calcinated material before the ball milling; S3) calcinated material after the ball milling, at (a) 300 °C and (b) 700 °C.

Figure 1a,b shows the different stages of nanoparticle production. HAP-based powders were obtained by calcination at 300 and 700 °C (samples nHAP_300_ and nHAP_700_, respectively). Different temperatures were chosen as previous studies showed this to be a significant parameter for the solubilization of the phosphorus present in the material and for the overall performance as a fertilizer. More specifically, at 300 °C, the presence of residual uncombusted carbon could have a beneficial effect; at 700 °C, on the other hand, the higher crystallinity level could be determinant.^51^

**Figure 2:**
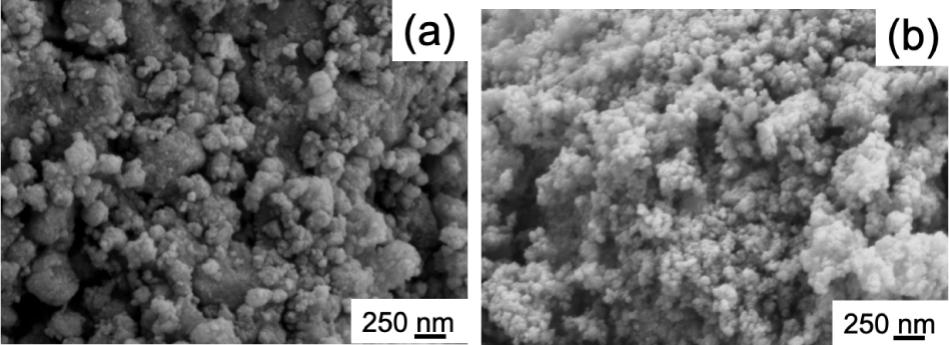
SEM images of a) nHAP_300_ and b) nHAP_700_ at 60 kX magnification.

**Figure 3:**
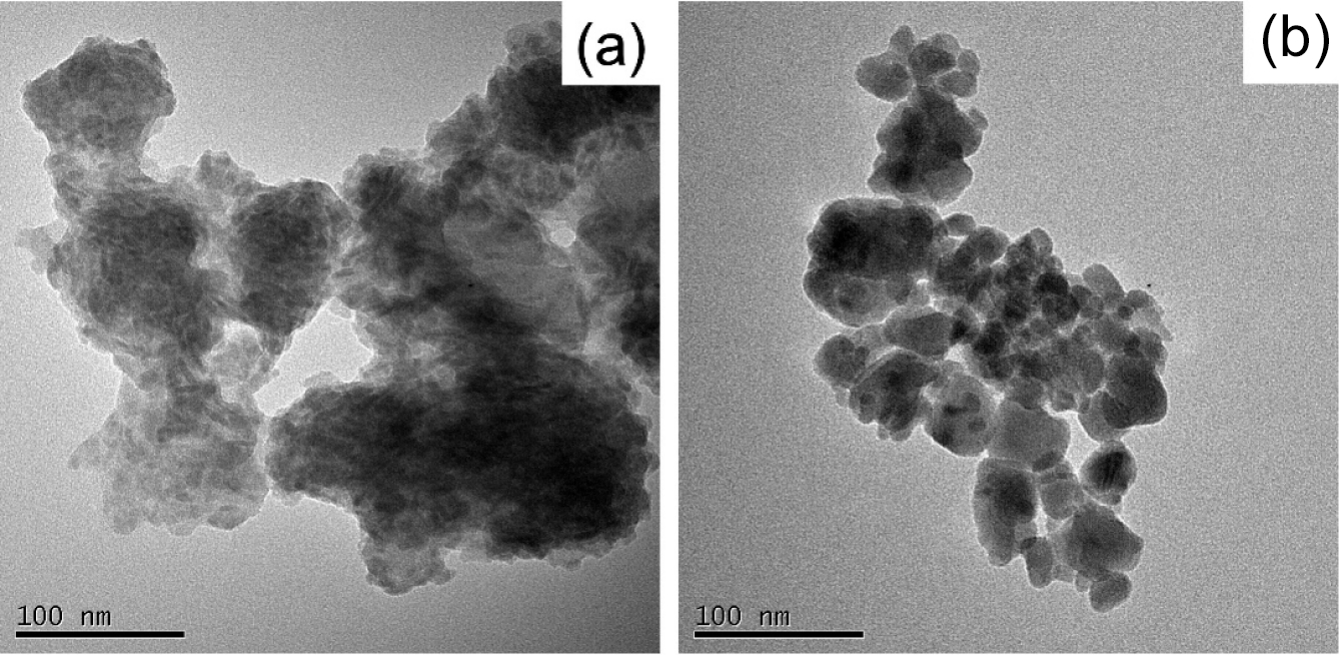
TEM images of a) nHAP_300_ and b) nHAP_700_.

To study the morphology of the HAP-based powders derived from the chicken bones, SEM analysis was performed; results are shown in Figure 2a,b for nHAP_300_ and nHAP_700_, respectively. Both nHAP particles are generally spherical. nHAP_300_ particles have a heterogeneous size distribution due to the presence of aggregates where the particles are held together by unburned carbon, which helps maintain the particles agglomerated. For nHAP_700_ powders, with a more uniform size distribution, a size on the order of 100 nm can be estimated. To have additional details on the size of the powders and possible aggregation, TEM microscopy was also considered (see Figure 3).

**Figure 4:**
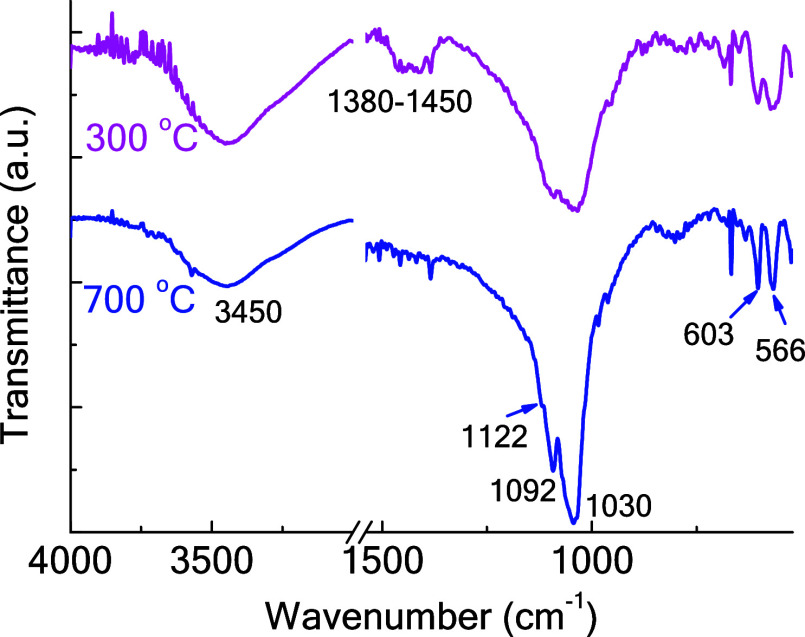
FTIR spectra for nHAP powders.

The image for sample nHAP_300_ (Figure 3a) confirms the particle agglomeration already observed in SEM; for sample nHAP_700_, on the other hand, the separate particles are visible, with their dimensions being on average just below 100 nm. Figure 4 shows the FTIR analysis of both powders.

It can be seen that the spectra have some similarities, but some differences are also present. The peaks in sample nHAP_700_ are sharper than those in nHAP_300_; this is due to the higher crystallinity resulting from the higher calcination temperature. In nHAP_300_, in agreement with the literature,^52^ the main peak associated with the PO_4_ group in HAP is detected at about 1030 cm^–1^, a very broad signal; considering nHAP_700_, the same peak is sharper, and it is possible to see other signals corresponding to PO_4_, at 1092 and 1122 cm^–1^ (a weak shoulder). The latter one is particularly important, as it belongs to β-Ca_3_(PO_4_)_2_ (β-TCP).^53^ This indicates that this phase is also present in the nHAP_700_ powder, although in a concentration lower than that of HAP. β-TCP formation can take place with thermal treatment when the Ca/P molar ratio is below the stoichiometric one for HAP (1.67); indeed, this is the case for both powders (see Table 2 for the elemental analysis) as the values are 1.50 and 1.47 for nHAP_300_ and nHAP_700_, respectively. Such a lower value is closer to the β-TCP stoichiometric ratio, i.e., 1.50; this favors the formation of such phase.

**Table 2:** Elemental content of nHAP_300_ and nHAP_700_^a^

	nHAP_300_	nHAP_700_
C (g·kg^–1^)	313.33 ± 2.84	3.80 ± 0.26
Ca (g·kg^–1^)	173.26 ± 16.92	357.07 ± 5.16
Fe (g·kg^–1^)	0.12 ± 0.07	0.27 ± 0.00
K (g·kg^–1^)	5.90 ± 0.35	12.60 ± 0.05
Mg (g·kg^–1^)	3.91 ± 0.16	8.11 ± 0.07
N (g·kg^–1^)	55.80 ± 1.51	0.60 ± 0.10
Na (g·kg^–1^)	2.76 ± 0.19	6.93 ± 0.35
P (g·kg^–1^)	88.34 ± 3.26	194.34 ± 2.89
Zn (g·kg^–1^)	0.18 ± 0.01	0.36 ± 0.00
Ca/P molar ratio	1.50	1.47

a Data are expressed in g/kg and as means of three replicates with the standard deviation. C and N contents were determined with a CHN analyzer, and Ca, F, K, Mg, Mn, Na, P, and Zn were determined with an ICP-OES instrument.

The peaks at 566 and 603 cm^–1^ also belong to the PO_4_ group, while the broad one at about 3450 cm^–1^ corresponds to the OH group. In the interval 1450–1380 cm^–1^, a broad signal is present in nHAP_300_, which belongs to the CO_3_ group; more specifically, the carbonate group substitutes the phosphate group in the HAP lattice, in the so-called B-type carbonated HAP.^54^ This signal is present due to the organic mass present in the bones, which was not completely removed at 300 °C; for the sample prepared at 700 °C, on the other hand, this peak is almost not visible due to the almost complete combustion. These data are in agreement with elemental analysis, which shows a much higher carbon content for nHAP_300_ than for nHAP_700_.

**Figure 5:**
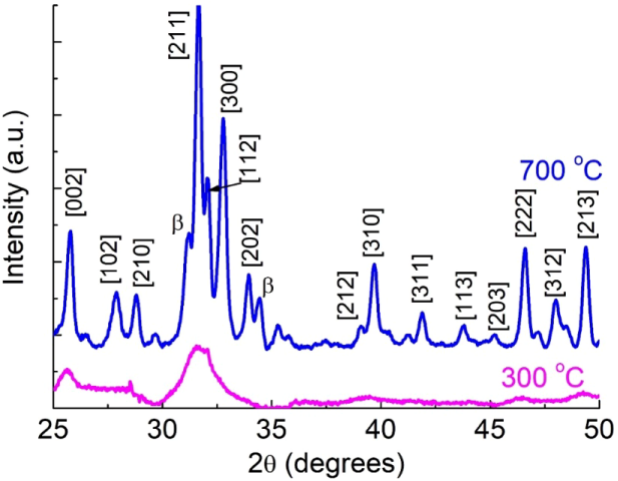
XRD patterns of the nHAP powders. Modified from Pilotto et al. (2024).

To confirm the FTIR findings, XRD patterns were also registered as reported by Pilotto et al. (2024),^42^ see Figure 5. The nHAP_300_ powder displays broad diffraction peaks, indicating low crystallinity due to the low-temperature treatment. Despite this, the characteristic hydroxyapatite peak at 31.7° is clearly observed. With an increase in calcination temperature from 300 to 700 °C, the diffraction peaks become sharper and more intense, due to the higher crystallinity of the material. The crystalline structure of nHAP_700_ closely matches hydroxyapatite, as verified by comparing its lattice parameters with those listed in the Joint Committee on Powder Diffraction Standards (JCPDS 09-0432) and illustrated in the graph. Additionally, secondary phases such as β-TCP (JCPDS N09-169) are identified in nHAP_700_ and marked with “β” in the figure. These results are in agreement with the FTIR data.

The crystallinity and crystallite size of nHAP_700_ powders were determined using eqs 1 and 2, respectively, yielding a crystallinity of 75% and a crystallite size of about 20 nm. This calculation was not performed for the sample nHAP_300_ due to its amorphous nature.

Considering again the elemental analysis, the two materials show other differences. Table 2 reports the results of ICP-OES and CHN analysis. nHAP_300_, due to the residual presence of organic matter, has a much higher content of carbon and nitrogen, 31.3% and 5.58%, respectively; for nHAP_700_, on the other hand, these elements are present at much lower concentrations, as they were almost completely burnt during the heat treatment, 0.38% and 0.06% for C and N, respectively. The removal of the organic matter in nHAP_700_ led to an increase in the proportion of the mineral part and consequently in the concentration of the other elements. An approximately 2-fold increase can be observed in the concentration of the other ions; more specifically, P content goes from 88.3 ± 3.25 g·kg^–1^ in nHAP_300_ to 194 ± 2.89 g·kg^–1^ in nHAP_700_.

Table 3 shows the values of the zeta potential (ZP) of the two nanometric HAP powders; they were measured in different media to understand the properties of the materials themselves and to show any possible change due to the interactions with the ions in solution. It is known, in fact, that ZP may change according to the ionic strength of the solution.^55^ When the powders were dispersed in distilled water, they both had negative potential values (−28 and −20.5 mV for nHAP_300_ and nHAP_700_, respectively); this is in agreement with the literature.^56^ In Luria–Bertani medium, on the other hand, the values are less negative, and there is almost no difference between the two samples; in fact, both have a ZP of about −12 mV. When NBRIP medium is employed, a significant difference can be seen between the two powders; in fact, nHAP_300_ has a negative ZP value (−7.71 mV) while nHAP_700_ has a ZP very close to zero (+0.06 mV). The more negative values of the samples prepared at 300 °C can be explained by considering the residual carbon present in the powder; the literature data, in fact, report that carbonaceous materials tend to have negative zeta potential values.^57^

**Table 3:** Zeta Potential Values for the nHAP Powders in Different Media (mV)

	nHAP_300_	nHAP_700_
Distilled water	–28 ± 5.25	–20.5 ± 4.08
Luria–Bertani medium	–12.3 ± 0.32	–11.6 ± 0.79
NBRIP medium	–7.71 ± 0.32	+0.06 ± 0.25

Surface area measurements were performed; nHAP_300_ and nHAP_700_ showed values of 25.16 and 20.55 m^2^/g, respectively. According to eq 3, an equivalent spherical diameter (*d*_BET_) of 125 nm was calculated for nHAP_300_ and 92 nm for nHAP_700_. A lower specific surface area and consequently a higher diameter with increasing temperature are due to the sintering of the powder, i.e., coalescence of smaller particles into larger ones through heating.^38^ The values of the surface area were comparable to those of other HAP samples of animal origin,^58^ although studies showed that significant differences could be observed according to the employed source.^59^

### 3.2 Phosphate Solubilization and Organic Acid Production by *P. alloputida*

**Figure 6:**
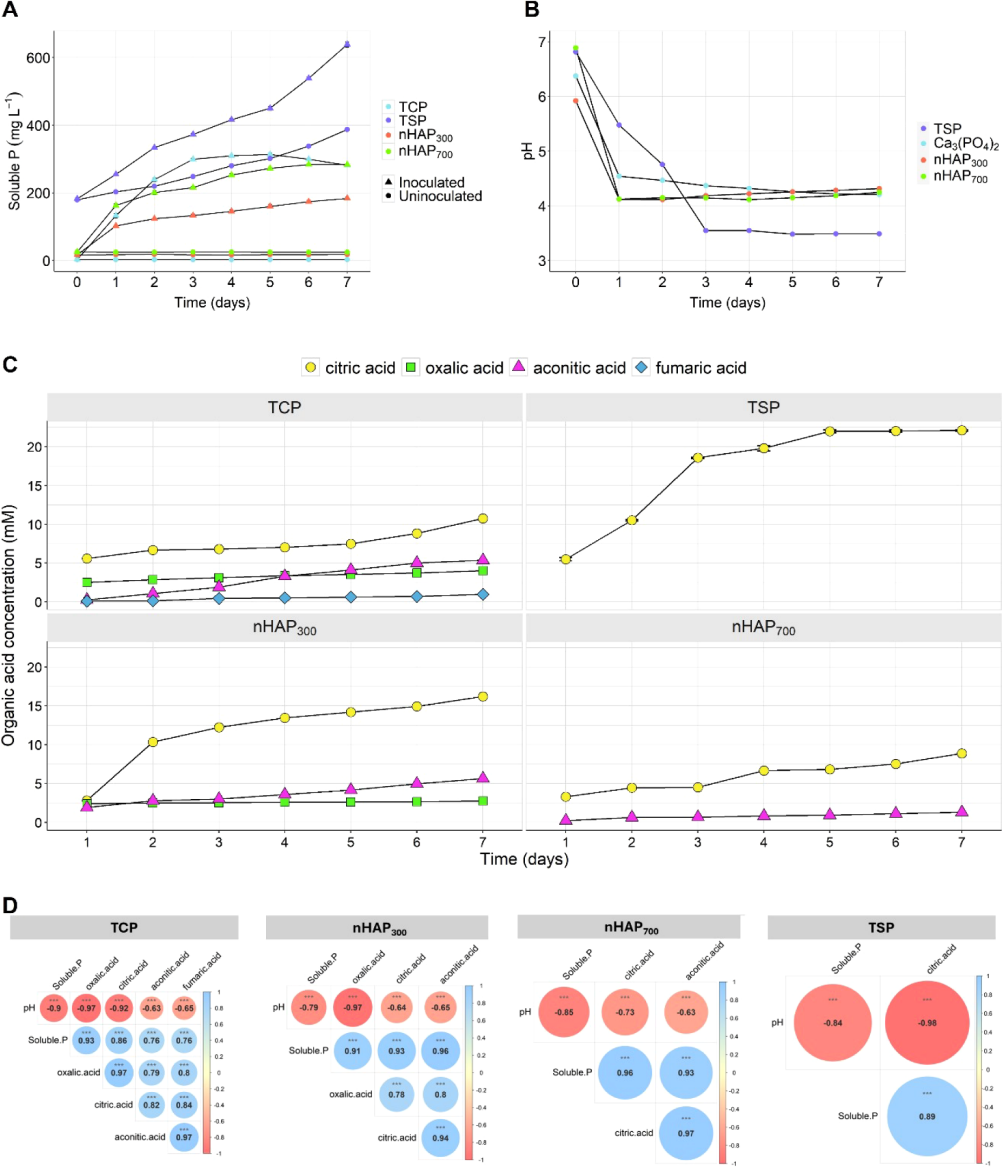
Mechanisms of P solubilization by *P. alloputida* DSM 6125 grown during 7 days in NBRIP liquid medium supplemented with four different P sources at 0.1% P (w/v). (A) P solubilization. (B) pH changes. (C) Organic acid production. (D) Pearson correlation matrix between solubilized P, pH values, and organic acids. Data are reported as means ± SE, *n* = 3. Statistical significance has been assessed by ANOVA, with Tukey’s posthoc test.

*Pseudomonas alloputida* DSM 6125 was tested for the solubilization of P from nHAP, both produced at 300 and 700 °C, in NBRIP medium in comparison with the model, insoluble P source tricalcium phosphate (Ca_3_(PO_4_)_2_, TCP) and the standard phosphate fertilizer used for crop production (triple superphosphate, TSP). As shown in Figure 6A, the samples added with TSP showed, as expected, from the beginning the highest concentration of soluble P. The inoculation with *P. alloputida* induced a further increase in soluble P concentration (up to 65% of the initial P added). On the other hand, the bacterial strain exhibited great ability in solubilizing TCP (up to 320 mg·L^–1^, i.e., 32% of the initial P added), followed by nHAP_700_ (up to 280 mg·L^–1^, i.e., 28% of the initial P added) and nHAP_300_ (up to 190 mg·L^–1^, i.e., 19% of the initial P added). The P solubilization rate from nHAP is in agreement with previous studies showing P solubilization rates ranging from 15% to 25% with *Bacillus megaterium* and *Sinorhizobium meliloti*, respectively.^60^ The soluble P measured in the inoculated media significantly increased during the incubation period in all P sources. In the case of TCP, *P. alloputida* showed its highest efficiency in solubilizing phosphate at a concentration of more than 100 mg·L^–1^ during the first two days and then gradually reduced until the fifth day when the highest amount of solubilized P was observed. However, during the last two days of incubation, a decrease in the amount of soluble P was observed. Regarding the phosphate solubilized in the media supplemented with nHAP_700_, the concentration was highest on the first day (162 mg·L^–1^) and gradually decreased until it reached the maximum and stable amount of soluble P equal to 280 mg·L^–1^ on the sixth day. The amount of phosphorus solubilized by *P. alloputida* in the medium supplemented with nHAP_300_ was significantly lower—by at least 1.5 times—compared to that in the other two phosphorus sources, although it continued to increase steadily until the end of the incubation period. On the other hand, in the uninoculated media, a basal but lower amount of soluble P was detected for the three P sources, possibly due to the autoclaving process. However, such amount did not change during the incubation period, indicating no bacterial growth in the media.

Since acidification is a key mechanism involved in P solubilization, pH variation was also monitored. Considering that the initial pH (before autoclaving) was adjusted to 7.0, it was possible to observe that the increased temperature during the sterilization process reduced the initial pH to 6.4 and 5.9 in the NBRIP medium supplemented with TCP and nHAP_300_, respectively. On the other hand, the initial pH of NBRIP medium supplemented with nHAP_700_ and TSP remained unaffected. After 1 day of incubation, the pH of inoculated media significantly decreased to 4.5 in TCP, while the pH in nHAP_300_ and nHAP_700_ dropped further to 4.1 (Figure 6B). In the subsequent days until the end of the incubation period, the pH did not present any significant variation. In the case of samples supplemented with TSP, the decrease in pH value was less steep, reaching the lowest values (pH = 3.5) at 3 days after inoculation as compared to the other treatments and remaining stable afterward. Conversely, the pH of uninoculated medium remained constant during the incubation period. To further characterize the solubilization process, the qualitative and quantitative profile of organic acids (OAs) released by *P. alloputida* was determined (Figure 6C), by analyzing the bacteria supernatant through HPLC. During the solubilization of TSP by *P. alloputida*, only citric acid was detected, which increased its concentration by about 5 times, up to 25 mM, during the experimental period. In the case of TCP solubilization, four main different OAs have been identified, namely citric, oxalic, aconitic, and fumaric acids, whose concentrations showed a differential variation during the incubation period. Over the period considered, the OA concentrations increased linearly, reaching different values at the end of the experiment. The most concentrated was citric acid, whose final concentration (10.7 mM) was doubled with respect to the initial one, followed by aconitic acid, which increased its concentration by about 20 times, up to approximately 5 mM (Figure 6C). Oxalic acid reached a final concentration of 4 mM, while fumaric acid was the least concentrated (0.97 mM), albeit its concentration increased by about 9 times with respect to the initial values detected (Figure 6C). Interestingly, when the source of insoluble P was represented by nHAP, an alteration in the qualitative and quantitative patterns of OAs released by *P. alloputida* was observed. When bacteria were treated with nHAP_300_, only citric, aconitic, and oxalic acids were detected. As in control conditions, citric acid reached the highest concentration (16.2 mM) at the end of the experiment, even though a steeper increase in the concentration was observed between days 1 and 2 (Figure 6C). When aconitic and oxalic acids are considered, the behavior was observed during the TCP solubilization. On the other hand, in the presence of nHAP_700_, only citric and aconitic acids were detected in the *P. alloputida* supernatants. However, the concentrations of these acids were lower compared to those of the other P treatments (i.e., TCP and nHAP_300_) (Figure 6C). The relationships among solubilized P, pH levels, and OAs were analyzed to investigate the underlying mechanism of P solubilization (Figure 6D). A significant negative correlation between solubilized P and pH suggests that acidification was a key process by which *P. alloputida* facilitated the dissolution of mineral P sources. Similarly, the negative correlation between pH and OA concentrations indicates that increased OA production by the bacteria contributed to the acidification of the NBRIP media. Furthermore, the positive correlation between OA concentrations and soluble P fraction (Figure 6D) suggests that the OAs produced by the bacteria contributed to the mobilization of the four different P sources tested.

### 3.3 Leached and Bioavailable P from the Soil Column Leaching Test

**Figure 7:**
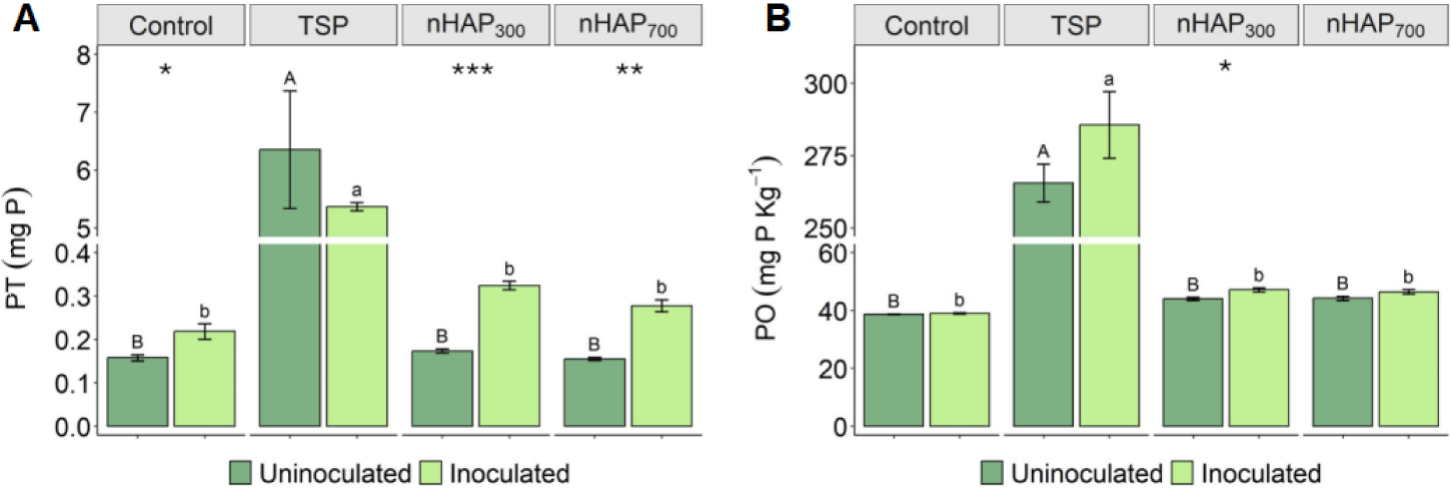
(A) Cumulative PT leached (mg) at the end of the soil column test (a) for the three fertilizers (TSP, nHAP_300_, and nHAP_700_) and the control (CTRL) treatment (no fertilization) with or without *P. alloputida* (PSB+ and PSB−) (mean value; *n* = 4; bars indicate standard error). (B) Bioavailable P (PO) in the soil (mg·kg^–1^) measured at the end of test (a) for the three fertilizers (TSP, nHAP_300_, and nHAP_700_) and the control (CTRL) treatment (no fertilization) with or without *P. alloputida* (PSB– and PSB+) (mean value; *n* = 4; bars indicate standard deviation). Different letters indicate significant differences (uppercase for ANOVA PSB– and lowercase for PSB+). Statistical significance of *t* tests is indicated with stars as follows: *p < 0.05, **p < 0.01, and ***p < 0.001.

For this part of the experimentation, the amount of phosphorus leached away during the waterings (PT) and the bioavailable phosphorus (PO) in the soil after the leachings were measured. Data indicate that, under the conditions tested, nanoparticles demonstrated lower mobility in soil compared to the conventional fertilizer, both with and without *P. alloputida*. Figure 7A represents the total P amount lost in the uninoculated and inoculated soil columns. These values are the results of the mean per treatment of the sum of all the quantities of P in milligrams lost in any leaching. The PT data identify relatively low amounts of P in the leachates of the CTRL, nHAP_300_, and nHAP_700_ treatments regardless of the presence of the microorganism, when compared to TSP. In uninoculated samples, the lowest mean value is recorded for the nHAP_700_ treatment (0.16 mg) with slightly higher, but not statistically different, mean values for CTRL and nHAP_300_ treatments. The average amount of PT by the columns treated with TSP (6.35 mg) is about 40 times higher than that of the other treatments. In the inoculated samples, the lowest mean value is reached by the CTRL treatment (0.22 mg) and not statistically different from nHAP_700_ and nHAP_300_ (0.28 and 0.32 mg, respectively). The average amount of dissolved P in the TSP columns (5.37 mg) is higher than the other treatments (from 16 to 25 times higher). The ANOVA identified a high significance due to the fertilization type (*p* < 0.001), no significance due to the inoculum, and no significance in the interaction of the two factors. About TSP, it is interesting to note that the value of PT decreased by about 25.11% in the presence of the microorganism.

However, it is worth noting that a pairwise comparison (Student’s *t* test) between inoculated and uninoculated samples among the same treatment showed significantly higher PT levels in PSB+ treated samples for CTRL (*p* = 0.035), nHAP_300_ (*p* < 0.001), and nHAP_700_ (*p* = 0.002). In these three treatments, the mean values are statistically higher than the PSB– ones, showing that *P. alloputida* induces a higher P dissolution from nHAP. This is confirmed by the ANOVA carried out for PT data considering only the treatments CTRL, nHAP_300_, and nHAP_700_. In this case, both the fertilization-type factor (FERT) and the PSB factor (*p* < 0.001) are significant, as well as the interaction FERT:PSB (*p* = 0.002), showing that the microorganism is able to enhance the P solubilization also in soil.

The PO data show relatively low quantities of bioavailable P in the soil when comparing the TSP treatments with the others, the former being approximately 6–7 times higher than the two others (Figure 7B). In the uninoculated samples, the lowest average data occur in the CTRL (38.67 mg·kg^–1^), with slightly, but not significantly, higher values for the nHAP_300_ and nHAP_700_ treatments (44.44 and 44.24 mg·kg^–1^). In the samples inoculated with *P. alloputida*, the lowest value was reported for the CTRL level (39.08 ppm), not significantly different from nHAP_300_ and nHAP_700_ treatments (47.16 and 46.49 mg·kg^–1^, respectively) compared to TSP, which showed the highest value in this case as well. The ANOVA data identified that the only significant factor is FERT (*p* < 0.001), and the *t* tests carried out for the comparison of the PSB factor for each level of the FERT factor showed that the only significance was for the nHAP_300_ level (*p* = 0.013). The ANOVA for PO data excluding the TSP data reported a high significance for both the FERT factor (*p* < 0.001) and the PSB factor (*p* = 0.001) and no significance for the interaction.

## 4 Discussion

HAP-based powders of the nanometric scale were successfully obtained from chicken bones. These results are in agreement with the literature, which reports nanometric HAP extracted from bones (residues of the food industry).^61^ Although the morphology of nHAP_700_ is more regular than nHAP_300_, both samples were tested for P solubilization; in fact, as mentioned in the previous section, the literature reports that in some cases, the presence of residual carbon could favor the solubilization.^51^ Our results, however, show that nHAP_700_ has better solubility than nHAP_300_; indeed, levels of solubilized phosphorus comparable to those of traditional TCP were achieved. It has to be highlighted, however, that HAP extracted from animal bones can have different characteristics according to the source of the bones. Moreover, in the cited study, the solubilization was performed without the use of the PBS strain. In the present work, the use of *P. alloputida* showed a significant contribution in enhancing P solubilization, as expected, and also significantly differentiated the extent of the solubilization between the two nanopowders. The better performance of nHAP_700_ could be explained by considering the presence of β-TCP shown by FTIR spectra and XRD diffraction patterns; this phase is, in fact, more soluble than HAP.^62^ Moreover, as NPs were not agglomerated, interaction with the bacterial strain may have been easier. Such higher solubilization for nHAP_700_ was registered despite nHAP_300_ having a higher surface area, which showed to be a less determinant parameter in the microorganism–NP interaction.

Previous experiments were performed on the use of PBS to solubilize HAP-based materials; it is difficult, however, to conduct a proper comparison because, as already mentioned above, the solubilization process may be affected by different parameters; HAP characteristics are surely determinant, but other factors such as the experimental conditions and the type of bacterial strain employed also play a very important role.^63^

In the present work, HAP derived from agrifood waste was studied, envisaging a recovery and valorization of these residues. Very few investigations were performed using this kind of HAP combined with PBS bacteria, with different and not easily comparable results;^64,65^ indeed, a more systematic study should be performed to better understand this topic.

As shown by DLS data, the ZP values of the NPs change significantly according to the medium; in fact, for both samples, the ZP values are less negative in culture media than in distilled water. More specifically, when the materials are suspended in the NBRIP medium, nHAP_700_ shows a ZP value very close to zero, while nHAP_300_ has a negative ZP value (about −8 mV). These differences between different media are reasonable, considering their very different compositions and the presence of ions, with consequent different interactions with the surface of the powders. As the solubilization experiments were performed in NBRIP, those values are the most important to explain the extent of the solubilization itself. The differential ability of *P. alloputida* to access and solubilize P from the different nHAPs might be, at least partially, explained by its ability to interact with the nanoparticles. Indeed, surface charge density is one of the parameters that is responsible for interactions and bacterial adhesion onto surfaces.^66^ Given that bacteria typically have a net negative charge due to carboxyl, amino, and phosphate groups on their cell wall surfaces,^67^ increased adhesion is frequently observed on positively charged surfaces.^66^ However, other reports indicate that some bacteria, in spite of their negative surface charge, can overcome electrostatic repulsion through surface structures like fimbriae.^68^ Additionally, lipopolysaccharide (LPS), a surface polymer found in Gram-negative bacteria (e.g., *P. aeruginosa* and *Escherichia coli*), can efficiently mediate the adhesion of bacteria to negatively charged surfaces.^69^ In addition, the decrease in the pH observed during the solubilization experiment could possibly alter the ionization of functional groups, therefore affecting the electrostatic forces involved in the bacterial interactions.

Several pieces of evidence demonstrate that PSB can mediate phosphorus solubilization from insoluble sources by lowering the pH and by releasing organic acids capable of chelating metals. This process induces phosphorus solubilization via ligand exchange reactions.^29,70−72^ As expected, the *P. alloputida* strain tested in this study demonstrated a strong ability to solubilize various sources of inorganic P, including nHAP synthesized at both 300 and 700 °C. This solubilization is related to an acidification mechanism and the release of OAs. However, the qualitative and quantitative analysis of the OA profiles indicated differential production depending on the P source, consistent with findings by other authors.^73^ In this context, it is worth mentioning that there is a substantial link between bacterial surface interactions and the regulation of the metabolome, which can result in the production and exudation of specific metabolites, OAs among them, that could play a pivotal role in the successful survival in different environmental niches.^74^ Specifically, with nHAP_700_, the bacteria produced citric and aconitic acid, whereas with nHAP_300_, *P. alloputida* also produced oxalic acid in addition to the other two acids. Interestingly, these findings align with previous observations that different *Pseudomonas* isolates (i.e., CBD35 and BWB 21) can solubilize insoluble P sources (e.g., rock phosphate) by releasing gluconic acid.^75^

The higher phosphate solubilization observed for nHAP_700_, despite its increased crystallinity, can be attributed to its positive zeta potential in the NBRIP medium, in contrast to the negative zeta potential of nHAP_300_. Organic acids released by *Pseudomonas alloputida*, such as citric, oxalic, and aconitic acids, are negatively charged due to their carboxylate groups. The positively charged surface of nHAP_700_ in this medium likely enhances its interaction with these organic acids, promoting a stronger electrostatic attraction between the negatively charged organic acid molecules and the positively charged HAP surface. This interaction facilitates more effective binding and subsequent chelation of calcium ions, destabilizing the hydroxyapatite structure and leading to enhanced phosphate solubilization.^76^ In contrast, nHAP_300_’s negative zeta potential could create electrostatic repulsion, limiting the interaction with negatively charged organic acids and, consequently, its phosphate solubilization efficiency.

The analysis of leachates has revealed a significant P concentration in samples collected from TSP columns, with values notably exceeding those observed in control, nHAP_300_, and nHAP_700_ columns by a factor of approximately 16–25. This outcome aligns with expectations, given TSP’s recognized characteristics as a water-soluble fertilizer. P solubilization can saturate soil’s P retention capacity, increasing its potential to move vertically through the soil profile. The current rates, timing, and methods of TSP application, combined with its propensity to release P rapidly, raise significant concerns about environmental impacts.^77^ Several studies in the literature have proposed strategies to mitigate the P loss associated with TSP fertilization. These approaches include efforts to slow down P release, such as the use of hydrochars^78^ or the application of coating polymers.^79^ Additionally, attempts have been made to substitute TSP with more environmentally friendly P sources.^80^ The use of natural HAP in the nanometric form proposed here may offer an alternative, as its low solubility^51^ appears to correspond to reduced leaching in this specific study. However, their agronomic benefits and environmental impact should be validated in long-term and field-scale studies.

Moreover, the TSP mobility in soil is highly correlated with soil texture. A study by Elliott et al. (2002) highlighted how P losses from TSP fertilization are correlated with soil type. Sandy soils have a poor capacity to retain TSP.^81^ This result is confirmed by another study by Xiong et al. (2018), where the authors compared the mobility in the soil of TSP and nHAP. They found higher mobility of the commercial fertilizer through the sand contrasted with the nanoparticles.^82^ The soil used in this experiment (Table 1) is a sandy clay loam soil, but it was enriched in sand to facilitate drainage. TSP characteristics and the type of soil could explain why P values for both types of nanoparticles are closer to the control untreated compared to TSP. Another reason could be attributed to the soil pH and the iron content. A study by Montalvo et al. (2015) proposed a percolation experiment similar to the one presented in this research. The authors analyzed leachates derived from columns filled with two acid soils, Andisols (pH = 5.30, Feox = 16.7 g·kg^–1^) and Oxisols (pH = 4.14, Feox = 4.14 g·kg^–1^), treated with TSP and synthetic nHAP. The experiment showed that the P mobility through the soil column depends heavily on the soil type, with Andisol showing a low P concentration in TSP columns and a high concentration in nHAP columns and Oxisol exhibiting the opposite pattern. In the authors’ opinion, this could be explained by considering the different iron oxide contents that can bind nHAPs.^83^ Concurrently, in the same work cited above, Xiong et al. (2018) filled soil columns with two types of soil, Ultisol (pH = 4.7) and Vertisol (pH = 8.2). In this case, the authors detected higher mobility of P from nHAPs in more acidic soil than in the Vertisol, where the P losses for any treatments are almost null.^82^ Our soil (Table 1) is subalkaline (pH = 7.6) and rich in iron (24.34 g·kg^–1^), which could explain the obtained results.

The impact of the microorganism was assessed based on the data provided in Section 3.2, revealing that *P. alloputida* effectively solubilized both types of nanoparticles in a nutrient-rich environment under optimal growth conditions. In the leaching experiment, *P. alloputida* induced varying increases in soil PO at the experiment’s conclusion: 1.04% for the CTRL, 11.08% for TSP, 6.58% for nHAP_300_, and 3.90% for nHAP_700_ treatments. However, statistical analysis revealed a significant difference only in the case of nHAP_300_. Notably, there are no known similar studies involving the same strain of phosphate-solubilizing bacteria at the time of writing; therefore, it is not possible to perform a proper comparison. One possible explanation could be that the absence of plants limited the efficacy of *P. alloputida*. Phosphate-solubilizing bacteria typically live in the rhizospheric soil, forming symbiotic relationships with plants.^84^ For this reason, further experiments with plants are necessary to investigate this hypothesis.

In conclusion, this study demonstrates that nHAPs synthesized from poultry waste at 700 °C possess properties that could be advantageous for agricultural use. With a phosphorus content of 19.4%, nHAP_700_ closely resembles TSP (at nearly 20%) in composition. Both types of nanoparticles exhibited reduced mobility in soil under our experimental conditions, resulting in lower P leaching compared with conventional TSP fertilizer, suggesting a potential advantage in specific scenarios. Moreover, using waste materials for nanoparticle synthesis offers a dual benefit: reducing environmental pollution associated with agricultural P leaching and reclaiming phosphorus from waste, thus supporting two key objectives of the European Green Deal. These biogenic nHAPs, by converting waste into a valuable nutrient source, present a promising alternative to traditional fertilizers. If future research confirms their efficacy in plant studies, field trials, and long-term assessments while ruling out toxicity for humans and the environment, nHAPs could transform agricultural practices by providing a sustainable, eco-friendly fertilizer option.
